# Complete and Incomplete Alcohol Sclerotherapy for Treatment of Symptomatic Hepatic Cysts: Comparison of Volume Reduction and Clinical Outcomes

**DOI:** 10.3390/jcm13051472

**Published:** 2024-03-03

**Authors:** Ran Kim, Jung-Suk Oh, Su-Ho Kim, Ho-Jong Chun

**Affiliations:** 1 Department of Radiology, Chung-Ang University Gwang Myeong Hospital, Chung-Ang University College of Medicine, Gwang Myeong 14353, Republic of Korea; rankim1001@gmail.com; 2 Department of Radiology, Seoul St. Mary’s Hospital, The Catholic University of Korea, Seoul 06591, Republic of Korea; lucidnature@naver.com (S.-H.K.); hojongchun@gmail.com (H.-J.C.)

**Keywords:** sclerotherapy, alcohol, hepatic cyst

## Abstract

**Background**: The purpose of this study was to compare the efficacy of incomplete alcohol sclerotherapy with complete treatment for hepatic cysts. **Methods**: From 2005 to 2021, a total of 80 patients (19 males, 61 females; median age 65 years; age range, 42–86 years) who underwent alcohol sclerotherapy for symptomatic benign hepatic cysts were enrolled and retrospectively reviewed. Complete treatment was defined as injecting 25–33% of the aspirated cyst volume with alcohol in 2–3 cycles, with a maximum of 100 mL per cycle. The overall volume reduction rate was compared between the complete and incomplete treatment groups. The response, based on cystic volume reduction, was classified as a complete regression (CR), near-complete regression (NCR), partial regression (PR), or no response (NR). CR and NCR were considered objective responses. Among 80 patients with 85 hepatic cysts, 26 patients with 29 hepatic cysts received incomplete treatment. **Results**: The overall volume reduction rate was not significantly different between the complete and incomplete treatment groups (94.39% vs. 95.47%, respectively, *p* = 0.623). The CR and NCR groups showed a significantly higher rate of symptom improvement than the PR and NR groups (*p* = 0.043). **Conclusions**: In conclusion, the efficacy of incomplete alcohol sclerotherapy was not inferior to that of complete treatment.

## 1. Introduction

The incidence of benign hepatic cysts has been reported as 2–18% in the general population [1]. Most benign hepatic cysts are asymptomatic and do not require treatment, but some may grow large enough to cause symptoms, eventually requiring treatment. The most common symptom is pain and abdominal distension, while other symptoms may include an epigastric mass, dyspepsia, vomiting, dyspnea, or even leg swelling and weight loss. Rarely, obstructive jaundice occurs due to large centrally located hepatic cysts compressing the hepatic hilum [2,3]. In the past, surgical treatments, such as liver resection, wide deroofing, and cystojejunostomy, were performed, but they were associated with considerable morbidity [4].

Advances in minimally invasive percutaneous treatment have made it possible to treat with simple aspiration or drainage of the cystic fluid. However, these procedures are ineffective and often lead to recurrence [5,6]. This is believed to occur because secretions from the epithelial cell lining in hepatic cysts prevent the obliteration of the cyst. Several authors suggest the use of sclerosant agents to induce further coagulation and necrosis of the cyst epithelium, thereby resulting in the definitive obliteration of the cyst [7,8,9,10]. Since the introduction of the use of alcohol as a sclerosing agent to treat symptomatic hepatic cysts in 1985 [11], alcohol has been the most frequently utilized sclerosing agent and its action mechanism is to disable the secretion of cystic fluid by destroying the cells lining the cystic cavity [12]. There are some studies using other sclerosing agents such as minocycline, tetracycline, doxycycline, polidocanol, and hypertonic saline [13,14,15,16]. The cyst volume reduction rate after alcohol sclerotherapy is reported to be higher than that of other sclerosing agents, at about 80–100% [12].

However, in actual clinical situations, there are patients who are very sensitive to alcohol or who show symptoms of alcohol intoxication. In these cases, alcohol sclerotherapy cannot be performed because of concerns about the symptoms of alcohol intoxication, or, even if the treatment is started, it cannot be completed and the procedure is abandoned. This study was prompted by concerns about the efficacy of incomplete treatment, which includes insufficient volumes of injected alcohol, insufficient alcohol exposure times, or both. The purpose of this study is to compare the efficacy of incomplete alcohol sclerotherapy with complete treatment for hepatic cysts.

## 2. Materials and Methods

### 2.1. Patients

Informed consent for sclerotherapy was obtained from all patients, and our Institutional Review Board approved this retrospective study. This study included 123 hepatic cysts that underwent alcohol sclerotherapy in our hospital from 2005 to 2021. Sixteen cysts, proven by cytology to be polycystic liver disease, parasitic cysts, and malignant cysts, were excluded. Seventeen cysts were excluded due to loss to follow-up or could not be identified in follow-up images. And 5 cysts whose symptoms were not specified were further excluded. Finally, 85 benign hepatic cysts in 80 symptomatic patients with available follow-up data were included. The baseline characteristics of all patients are presented in Table 1. The main indication for treatment was abdominal discomfort and increasing abdominal girth.

### 2.2. Data Collection

All patients were evaluated retrospectively via review of their electronic chart, including radiologic studies after the procedure and medical reports. The total amount of aspirated cyst volume before alcohol injection, the amount of injected alcohol, the changes in symptoms, complications, number of sessions, and reasons for incomplete treatment were collected.

One experienced interventional radiologist reviewed computed tomography (CT) images. The location of the cyst was collected, the three maximal orthogonal cyst diameters, d1, d2, and d3, were measured, and the estimated cyst volume was calculated using the ellipsoid volume formula: volume = d1 × d2 × d3 × 0.523. Fluid re-accumulation immediately following aspiration sclerotherapy is a well-documented phenomenon. Therefore, the most recent CT image was utilized to calculate the estimated follow-up cyst volume.

The response according to the cystic volume reduction was classified as a complete regression (CR) when the cyst disappeared, as near-complete regression (NCR) when the volume reduction rate was greater than 90%, as partial regression (PR) when the volume reduction rate was between 50% and 90%, and as no response (NR) when the volume reduction rate was less than 50%.

### 2.3. Technique

The procedure was performed by one of four fellowship-trained interventional radiologists with an average of 20 years in practice in interventional radiology suites. The patient was placed in the supine position on an angiographic table. Under ultrasound guidance, the cyst was punctured with an 18 G 20 cm Chiba needle (Cook Medical, Bloomington, IN, USA). A guidewire was inserted and 8.5 French pigtail catheter (Cook Medical, Bloomington, IN, USA) was introduced through the guidewire. After complete aspiration of the cyst, contrast material was injected with fluoroscopy to exclude leakage into the peritoneal cavity or communication with vasculature or the biliary tree. And, then, 99% alcohol was injected in an amount of 25–33% of the aspirated cyst volume; the total amount of alcohol should not exceed 100 mL. The patient’s position was changed from supine to right, left lateral decubitus, and prone every 15 min, to achieve proper contact between the alcohol and cyst wall. After 60 min, all alcohol was re-aspirated and the catheter was naturally drained. The same protocol was repeated when additional sessions were needed; otherwise, the catheter was removed. Only the first procedure was included in this analysis when the patient underwent two or more sclerotherapy procedures for the same cyst.

**Figure 1:**
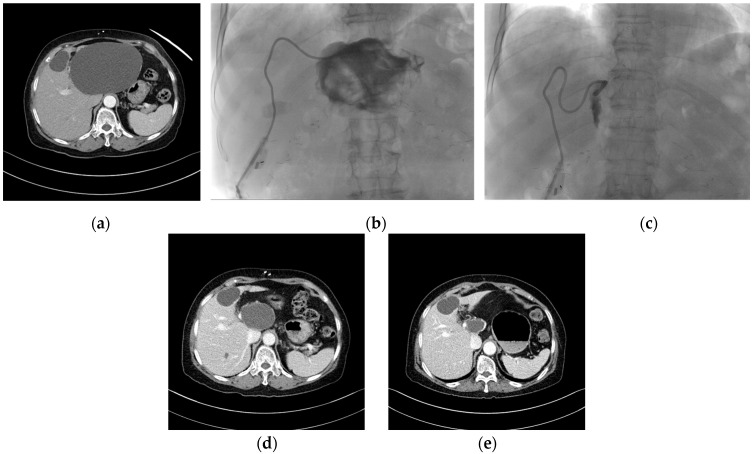
Case of incomplete alcohol sclerotherapy and natural course after treatment. (**a**) An enhanced CT scan performed on a 77-year-old woman presenting with early satiety revealed a large cyst measuring 14 × 10 × 13 cm maximum orthogonal cyst diameter in the left liver. The patient had a history of alcohol sensitivity. (**b**) After natural drainage of 1000 mL over the course of one day, tubography was performed. The cystic cavity was found to be collapsed, and contrast infusion and regurgitation proceeded smoothly. The planned injection of 100 mL of 99% alcohol was then initiated. However, at the time of injection of 40 mL, severe symptoms of alcohol intoxication manifested, which led to an immediate discontinuation of the injection and removal of the alcohol. (**c**) The next day, 18 mL of drainage was observed, so a second alcohol sclerotherapy was attempted, but severe pain occurred again when another 30 mL of alcohol was injected, so the alcohol was immediately removed. In the final tubography, the cystic cavity was found to be even more collapsed than before. Natural drainage was performed for 6 h, with minimal fluid drainage leading to the removal of the catheter. (**d**) CT scan performed after 6 months revealed the cystic cavity, measuring 6 × 5 × 6 cm in size. (**e**) CT scan performed after 12 months revealed that the size of the cyst was smaller (2 × 4 × 3 cm) without repeated sclerotherapy, and the cyst had shrunk to 96% of its pre-treatment size at 12 months.

Incomplete treatment was carried out for patients who were very susceptible to alcohol or who developed severe alcohol intoxication symptoms such as abdominal pain or epilepsy. Incomplete treatment was defined as follows: (1) the exposure time of alcohol was insufficient; (2) the injected volume of alcohol was less than 10% of the aspirated cyst volume (Figure 1).

### 2.4. Statistical Analysis

Statistical analysis was carried out per cyst. X2 and paired *t*-test were used to compare variables. The predictors of clinical outcomes were multivariably analyzed with a logistic regression test. Results were considered statistically significant if *p* < 0.05.

## 3. Results

A total of 80 patients (19 males and 61 females) aged 42–86 (median 65) years underwent sclerotherapy with alcohol for a total of 85 hepatic cysts. Among the patients, one had 4 treated hepatic cysts, and two had 2 treated hepatic cysts. Of these, 54 patients with 56 cysts received treatment following the routine protocol of alcohol sclerotherapy, while 26 patients with 29 cysts underwent incomplete treatment. The reasons for incomplete treatment were severe pain (*n* = 13), alcohol susceptibility (*n* = 12), and epilepsy (*n* = 1). Twenty-two patients underwent alcohol sclerotherapy with less than 10% of the aspirated cyst volume, and alcohol was re-aspirated immediately after instillation in two patients. Additionally, two patients received insufficient alcohol injection both in terms of volume and time. Seventy-eight patients underwent pre-treatment imaging using CT, while two patients had MRI. For post-treatment follow-up, all patients had CT scans. The mean follow-up period was 232 days.

The median aspirated volume of the 85 hepatic cysts was 500 mL. In the complete group, the median aspirated cyst volume was 500 mL with a range of 25–7200 mL. In the incomplete group, the median aspirated cyst volume was 455.5 mL with a range of 260–2550 mL. The median estimated pretreatment cyst volume of the 85 hepatic cysts was 506 mL. In the complete group, the median estimated pretreatment cyst volume was 506 mL with a range of 39–4351 mL. In the incomplete group, the median estimated pretreatment cyst volume was 468 mL with a range of 198–2240 mL. The median estimated post-treatment cyst volume of 85 hepatic cysts was 25 mL. In the complete group, the median estimated post-treatment cyst volume was 25 mL with a range of 0–4787 mL. In the incomplete group, the median estimated post-treatment cyst volume was 25 mL with a range of 0–377 mL. The mean injected alcohol volume was 54.9 mL. In the complete group, the mean injected alcohol volume was 65.8 mL, and in the incomplete group, the mean injected alcohol volume was 33.8 mL.

CR was achieved in 11 cysts (12.9%), NCR was achieved in 59 cysts (69.4%), and PR was achieved in 14 cysts (16.5%). One cyst showed no response. The CR and NCR group were considered as objective response groups, with approximately 82% of the cysts showing an objective response. The mean volume reduction rate of the 85 hepatic cysts was 94.76%. In the complete and incomplete treatment groups, the mean volume reduction rates were 94.39% and 95.47%, respectively, and these results were not significantly different (*p* = 0.623; Table 2). Additionally, the response based on cystic volume reduction was not significantly different between the complete and incomplete treatment groups (14.3%, 67.9%, 16.1%, and 1.8% and 10.3%, 72.4%, 17.2%, and 0% in CR, NCR, PR, and NR, respectively, *p* = 0.845; Table 2).

Several clinical variables were compared between the CR + NCR group and the PR + NR group to identify which variables influenced the objective response. The volume of the initially aspirated cyst, whether the cyst volume was greater than 1000 mL, the number of sessions, and the volume of injected alcohol were not found to be related to the objective response. The only statistically significant difference was observed in symptom improvement, which was higher in the CR + NCR group compared to the PR + NR group (*p* = 0.043, Table 3).

A multivariate analysis revealed that age, sex, and cyst location were not associated with the objective response. Additionally, the original cyst volume, number of sessions, and volume of injected alcohol were not associated with objective response (Table 4).

Four patients (4/80, 5%) experienced complications, including cyst rupture, cyst infection, bleeding, and transient drop in blood pressure. These side effects were managed with conservative treatment and resolved without any sequelae.

## 4. Discussion 

We defined our routine protocol (injection of an amount of 25–33% alcohol (not exceeding 100 mL) of the aspirated cyst volume in 2–3 cycles) based on previous studies and designed this study to investigate which factors influence the radiological and clinical response compared to the incomplete treatment group. Our result shows that there are no significant differences in overall volume reduction rate in the responses between the complete and incomplete treatment groups. In addition, it has been demonstrated that there was no significant difference in the original cyst volume, the number of sessions, and volume of injected alcohol between the CR + NCR group and the PR + NR group. These results are somewhat consistent with the findings of previous studies. 

Alcohol was used as a sclerosing agent in various ways across the studies, with variations in the duration and frequency of alcohol exposure, as well as the concentration and volume of the solution used. The number of aspiration sclerotherapy sessions also varies depending on the size and location of the cyst, as well as the patient’s response to the treatment. Some researchers [17,18] have used a multi-session approach, with intervals of 12 h to 2 days between each session, and have reported that this technique yields better outcomes than a single injection of a sclerosing agent in terms of reducing the recurrence rate of hepatic cysts. Moreover, larger cysts may require multiple sessions over a period of weeks or months to achieve the desired result. Conversely, other studies have reported positive results with a single session of alcohol sclerotherapy [8,10,19,20,21,22,23], which is sufficient to reduce the size of the cyst or even achieve complete resolution, with fewer risks. The duration of exposure to the sclerosant also varies widely, with reports ranging from 10 min to 4 h [10,19]. In addition, the amount of alcohol injected after aspiration ranges from 10% to 50% of the volume of the cyst, with the maximum recommended dose ranging from 100 to 200 mL [10,18,19]. Interestingly, most published studies investigating sclerotherapy using different protocols consistently report satisfactory results in terms of cyst volume reduction. Combining the results of previous reports and our studies, we can assume that the use of alcohol itself is more important for effective cystic volume reduction and is not greatly affected by the volume of alcohol or the time of alcohol instillation. However, more comparative studies on a larger scale are needed to validate this hypothesis.

Complications related to alcohol have been reported after alcohol sclerotherapy, ranging from minor alcohol intoxication symptoms, such as flushing, nausea and vomiting, skin rash, and deep sleep, to major symptoms such as severe abdominal pain, epilepsy, and decreased blood pressure [8,10,19,22]. Yang et al. [10] revealed that the mean blood alcohol concentrations increased over time up to 4 h after alcohol instillation. Alcohol intoxication has been reported more frequently in studies with higher doses of alcohol or longer duration times [24]. Therefore, patients with symptoms of alcohol intoxication often do not complete the regular protocol time and re-aspirate alcohol earlier, or patients with alcohol sensitivity may use a lower volume of alcohol. However, in clinical practice, patients who are highly sensitive to alcohol may still require cyst volume reduction due to severe symptoms. Our results demonstrate an effective reduction in cyst volume, even in cases where sclerotherapy was incomplete. This suggests that patients with severe alcohol intoxication can still achieve favorable clinical outcomes with incomplete alcohol sclerotherapy.

There are several limitations in this study. First, the data were obtained retrospectively from a single center. Second, the assessment of symptom relief was relatively subjective and did not use a grading scale or score system. Finally, there is a difference in the number of cysts between the complete and incomplete treatment groups.

In conclusion, the efficacy of alcohol sclerotherapy was not significantly inferior to that of the complete treatment group, even when the amount of alcohol or the application time was insufficient. Therefore, patients who are highly sensitive to alcohol can still undergo safe and effective treatment with incomplete sclerotherapy (small amount of alcohol and short alcohol installation time).

## Figures and Tables

**Table 1:** Baseline characteristics of the patients.

Age, years	65 (42–86) *
Sex; Male/female	19/61
Patient symptom	
Abdominal discomfort	38 (44.7)
Increasing abdominal girth	31 (36.5)
Abdominal pain	10 (11.8)
Early satiety	4 (4.7)
Dyspnea	1 (1.2)
Nausea and vomiting	1 (1.2)
Cyst location; right lobe/left lobe	60/25
Number of sclerotherapy sessions	1.5 (1–4) *

Values are the number of patients, with percentages in parentheses. * Median with ranges in parentheses.

**Table 2:** Response comparison between complete versus incomplete treatment group.

	Complete Treatment	Incomplete Treatment	
Volume reduction (%)	94.39 ± 10.77 *	95.47 ± 6.47 *	p = 0.623
Response			p = 0.845
CR	8 (14.3%)	3 (10.3%)	
NCR	38 (67.9%)	21 (72.4%)	
PR	9 (16.1%)	5 (17.2%)	
NR	1 (1.8%)	0 (0%)	

CR, complete regression; NCR, near complete regression; PR, partial regression; NR, no response. Values are the number of patients, with percentages in parentheses. * Mean with standard deviation.

**Table 3:** Multivariate analysis according to the response.

	Complete/Near Complete Regression (*n* = 69)	Partial Regression/No Response (*n* = 16)	*p*-Value
Age (year) *	65.78 ± 10.24	65.31 ± 6.97	0.862
Sex, female	56 (81.2%)	10 (62.5%)	0.106
Location, right	46 (66.7%)	14 (87.5%)	0.099
Initially aspirated cyst fluid (mL) *	733.65 ± 938.46	963.75 ± 955.93	0.381
Original cyst volume (mL)			0.586
Huge (≥1000 mL)	17 (24.6%)	5 (31.3%)	
Not huge (<1000 mL)	52 (75.4%)	11 (68.8%)	
No. of sessions			0.203
1~2	36 (52.1%)	11 (68.4%)	
3~4	33 (47.8%)	5 (31.3%)	
Volume of injected alcohol *	52.46 ± 29.1	65.31 ± 32.94	0.124
Symptom change	66 (95.7%)	13 (81.3%)	0.043

Values are the number of patients, with percentages in parentheses. * Mean with standard deviation.

**Table 4:** Predictors of objective response: multivariate analysis.

	Odds Ratio	95% CI	*p*-Value
Age	0.035	0.939–1.076	0.878
Male	0.704	0.822–12.969	0.093
Location, right	0.857	0.710–20.399	0.119
Original cyst volume (Huge (≥1000 mL))	0.866	0.175–5.219	0.959
No. of sessions (12/34)	0.699	0.088–1.364	0.130
Volume of injected alcohol	0.012	0.965–1.010	0.268

## Data Availability

Data are available on request.
